# Real-Life Effectiveness and Safety of Bimekizumab in Plaque Psoriasis Involving Difficult-to-Treat Areas: A 52-Week, Retrospective Real-World, Single-Center Study

**DOI:** 10.3390/jcm14207412

**Published:** 2025-10-20

**Authors:** Matteo Bianco, Francesco D’Oria, Gioele Ghezzi, Luciano Ibba, Sara Di Giulio, Mario Valenti, Antonio Costanzo, Alessandra Narcisi, Luigi Gargiulo

**Affiliations:** 1 Dermatology Unit, IRCCS Humanitas Research Hospital, Rozzano, 20089 Milan, Italy; matteo.bianco@humanitas.it (M.B.); francesco.doria@humanitas.it (F.D.); gioele.ghezzi@humanitas.it (G.G.); luciano.ibba@humanitas.it (L.I.); sara.digiulio@humanitas.it (S.D.G.); mario.valenti@hunimed.eu (M.V.); antonio.costanzo@hunimed.eu (A.C.); alessandra.narcisi@humanitas.it (A.N.); 2 Department of Biomedical Sciences, Humanitas University, Pieve Emanuele, 20089 Milan, Italy

**Keywords:** bimekizumab, nail psoriasis, scalp psoriasis, palmoplantar psoriasis, genital psoriasis, interleukin 17, psoriasis

## Abstract

**Background:** Psoriasis is a chronic inflammatory disease that frequently affects difficult-to-treat areas such as the scalp, nails, genitalia, and palms/soles, with significant physical and psychological burden. Bimekizumab, a monoclonal antibody targeting both interleukin (IL)-17A and IL-17F, has shown rapid and durable efficacy in clinical trials, but real-world data in these subgroups remain limited. **Methods**: We performed a 52-week, single-center retrospective study including patients with psoriasis involving at least one difficult-to-treat area. Effectiveness was assessed using site-specific Physician’s Global Assessment (sc-PGA, f-PGA, sPGA-G, pp-PGA). The primary endpoint was the proportion of patients achieving a PGA 0/1 (clear or almost clear). Safety data were collected at each visit. **Results:** Eighty-five patients were included (61.8% male; mean age 48.1 years; mean Body Mass Index (BMI, 26.9 kg/m^2^). Difficult-to-treat areas involved were the scalp (70.6%), nails (41.2%), genitalia (27.1%), and palms/soles (24.7%). At week 52, sc-PGA 0/1 was achieved in 90.6% of patients, sPGA-G 0/1 in 81.3%, f-PGA 0/1 in 66.7%, and pp-PGA 0/1 in 87.5%. Mean PGA values progressively decreased across all sites. The most common adverse event was oral candidiasis (11.8%). **Conclusions:** Bimekizumab showed rapid, sustained, and clinically meaningful improvement across all difficult-to-treat areas with a favorable safety profile.

## 1. Introduction

Psoriasis is a chronic, immune-mediated systemic disease that predominantly affects the skin and the joints, with a global prevalence estimated at approximately 2–3% of the general population [1]. From a clinical standpoint, it is most commonly characterized by well-demarcated, erythematous, and scaly plaques typically located on the extensor surfaces of the body, such as the elbows and knees, as well as on the lumbosacral region. However, the disease can also involve other anatomically and functionally critical areas, including the scalp, face, palms and soles, genital region, and nails. When these sites are affected, the impact on patients is often disproportionately high compared with the extent of skin involvement, which can sometimes even be very limited. The involvement of these regions is frequently associated with increased physical discomfort and functional limitations, along with marked psychosocial consequences such as embarrassment, stigmatization, social withdrawal, and impaired sexual health. Altogether, these aspects significantly contribute to the overall burden of disease [2].

Moreover, these areas tend to be particularly recalcitrant to treatment. Therefore, these sites deserve the reputation of being difficult-to-treat areas [3], thus representing a challenge to clinicians and patients. Indeed, the skin in these regions is typically thinner, more sensitive, and prone to mechanical stress. All these features contribute to make skin more prone to irritation, thus limiting both the use of topical treatments and patients’ adherence [4]. Although conventional immunosuppressive therapies, such as cyclosporine, methotrexate and acitretin, can be valid options as a first approach, their profile of adverse events and the need for close laboratory monitoring limit the possibility of long-term courses with these therapeutic agents [5]. Besides offering safer options, biological therapies are growingly emerging as effective and paradigm-shifting therapies, when it comes to managing psoriatic involvement of difficult-to-treat areas [6,7].

In recent years, the advent of biologic drugs has radically transformed the treatment landscape of moderate-to-severe psoriasis [8]. Among these, inhibitors of the interleukin (IL)-23/IL-17 axis have demonstrated significant efficacy and safety profiles.

The IL-17 interleukin (IL-)17 family plays a pivotal role in skin homeostasis, as well as being responsible of the defense against extracellular bacteria and fungi [9]. Moreover, IL-17 has been deeply studied due to its role in immune-mediated inflammatory diseases, such as psoriasis [10].

IL-17 family includes different isoforms, comprising IL-17A, IL-17B, IL-17C, IL-17D, IL-17E (also known as IL-25), and IL-17F [11], each with distinct but overlapping roles in immune regulation [11]. Each isoform exerts its functions in a homodimeric form.

IL-17A is a widely recognized key driver of psoriatic inflammation, as it promotes keratinocyte proliferation, neutrophil homing, and amplification of inflammatory pathways in the skin [12]. This is the rationale of the development of highly effective agents, such as secukinumab and ixekizumab [13].

On the other hand, there is emerging evidence suggesting that IL-17F, which shares structural similarities with IL-17A and can form heterodimers with it, is abundantly expressed in psoriatic lesions, where it contributes to disease chronicity and recurrence [14]. In fact, IL-17F levels have been shown to be even higher than IL-17A in lesional skin [15]. This brought clinicians to hypothesize that IL-17F blockade may offer additional therapeutic benefits, particularly in patients who failed to respond or experienced suboptimal responses to selective IL-17A inhibitors [16].

Since patients’ quality of life is markedly affected by psoriatic involvement of the aforementioned areas, a rapid-acting therapy is particularly important in this group of patients. IL-17 inhibitors (IL-17i) are widely recognized to meet this need for rapidity, as they have been shown to be faster than IL-23 inhibitors (IL-23i) in reaching the desired outcomes [17]. In clinical practice, the speed of response often represents a key determinant of patients’ satisfaction, in particular in cases where visible lesions cause social stigma or functional impairment.

Bimekizumab is a humanized monoclonal antibody that exerts its effects through dual inhibition of both IL-17A and IL-17F. This results in deeper and more sustained clinical responses compared to IL-17A blockade alone [18].

Phase III trials such as BE VIVID, BE READY, and BE RADIANT demonstrated excellent efficacy of bimekizumab in terms of achieving PASI 90 and PASI 100, as well as a rapid onset of action and durable control of disease activity [19]. Notably, subsequent subgroup analyses performed on these trials and real-world studies highlighted remarkable results in treating psoriasis involving difficult-to-treat areas, such as the nails, scalp, and genitalia [20].

Moreover, the safety profile of bimekizumab has been demonstrated to be favorable, with most adverse events represented by candidiasis reports, which turn out to be easily manageable in clinical practice [21].

Despite the robust data from randomized controlled trials (RCTs), real-world evidence remains essential to confirm both the effectiveness and tolerability of bimekizumab in broader and more heterogeneous patient populations, who are usually excluded from RCTs. Results from observational studies may provide valuable insights into actual drug performance in everyday clinical practice, where patients’ characteristics frequently differ from those in the controlled settings of RCTs. Indeed, the strict inclusion criteria of RCTs exclude individuals with multiple comorbidities or atypical clinical presentations, which, by contrast, are quite common in real-world settings.

Given the highly unmet needs in patients with psoriasis involving difficult-to-treat areas and the promising profile of bimekizumab as a dual IL-17A/IL-17F inhibitor, our study aims to assess the real-world effectiveness and safety of this drug in this specific subpopulation. In particular, we aimed to evaluate whether bimekizumab improves site-specific PGA for difficult-to-treat areas in psoriasis patients after 52 weeks of treatment in a real-world clinical setting.

## 2. Materials and Methods

We conducted a 52-week, single-center retrospective study aimed at evaluating the effectiveness of bimekizumab in the treatment of patients with psoriasis with significant involvement of at least one difficult-to-treat area (genital, scalp, nail, or palmoplantar region). Patients were treated at our Dermatology Unit from 1 January 2024, to 31 May 2025. All patients received bimekizumab according to the Italian version of the European Guidelines for the Clinical Management of Chronic Plaque Psoriasis. Bimekizumab was also administered in compliance with the Summary of Product Characteristics. Before starting bimekizumab, patients were screened for viral hepatitis, HIV, and latent tuberculosis, in accordance with current guidelines. During the 52-week follow-up period, routine laboratory examinations were performed, based on patients’ individual characteristics, as per standard clinical practice. Those patients treated with bimekizumab for plaque psoriasis who did not have any lesions of the aforementioned sites were not included.

Clinical data were analyzed at treatment initiation (baseline), week 16, week 32, and week 52. At the first dermatological visit, demographic data, previous therapies, general and cardiometabolic comorbidities, clinical characteristics, and disease duration were recorded. To assess treatment effectiveness at the prespecified endpoints, the following measures were evaluated: scalp-specific Physician’s Global Assessment (sc-PGA), fingernail PGA (f-PGA), static Physician’s Global Assessment of Genitalia (sPGA-G), palmoplantar PGA (pp-PGA), and Psoriasis Area and Severity Index (PASI). Patients were included if they had at least one site-specific PGA of 2 or higher. All adverse events (AEs) reported during the observation period were documented, including those leading to treatment discontinuation.

The primary endpoint was the proportion of patients achieving a site-specific PGA score of 0/1 (clear or almost clear) at each follow-up time point. The secondary endpoints were the mean reduction in site-specific PGA scores over time for each difficult-to-treat area, the proportion of patients achieving a reduction in PASI from baseline of at least 75% (PASI75), 90% (PASI90), and 100% (PASI100), and the proportion of patients achieving an absolute PASI ≤ 2 (PASI ≤ 2) at the respective time points. Continuous variables were expressed as mean ± standard deviation (SD), and categorical variables as absolute numbers and percentages. Normality of distribution was assessed for each continuous dataset using the Shapiro–Wilk test. For within-patient comparisons of mean site-specific PGA values between baseline and follow-up timepoints (weeks 16, 32, and 52), paired statistical tests were applied: paired Student’s *t*-test when normality was confirmed, and the Wilcoxon signed-rank test when normality was not met. For comparisons between independent groups (e.g., bio-naïve vs. bio-experienced patients), unpaired *t*-tests or Mann–Whitney U tests were used depending on data distribution.

Achievement of PGA 0/1 (clear or almost clear) at each time point was reported descriptively as the number and percentage of patients per subgroup. These values were not analyzed as formal binary outcomes with inferential testing, but rather presented to provide a descriptive overview of clinical response in each difficult-to-treat area. *p*-Values were therefore applied exclusively to the analyses of continuous mean PGA reductions over time. Exact *p*-values are reported whenever possible. STATA/SE 17.0 software (StataCorp. 2025. *Stata Statistical Software:* tataCorp LLC.: College Station, TX, USA) was used to conduct the data analysis.

Due to the retrospective nature of data collection, not all parameters were consistently recorded at each follow-up visit. In these cases, missing values were not imputed. Moreover, not all patients had completed the 52-week follow-up period at the time of data cutoff, since they started the treatment at different times. The only treatment discontinuations reported were those due to adverse events, which are described further. All patients provided informed consent for the collection of clinical data within routine practice, in accordance with applicable regulations on patient privacy. The study was conducted in accordance with the principles of the 1964 Declaration of Helsinki and its subsequent amendments.

## 3. Results

A total of 85 patients with psoriasis involving at least one difficult-to-treat area were included in the study. Demographic and clinical characteristics are summarized in Table 1. Sixty-one patients (71.8%) were male, with a mean age of 48.1 years (SD 16.8). The mean body mass index (BMI) was 26.9 kg/m^2^ (SD 5.8), and 31 patients (30.6%) had at least one cardiometabolic comorbidity (arterial hypertension, obesity, type II diabetes mellitus, hypercholesterolemia, and cardiovascular diseases). Eight patients (9.4%) had a concomitant diagnosis of psoriatic arthritis (PsA). Twenty-six patients (30.6%) had previously received at least one biological therapy (bio-experienced patients). The mean baseline PASI was 13.2 (SD 10.2).

Regarding the involvement of difficult-to-treat areas, 60 patients (70.6%) had scalp involvement, 23 patients (27.1%) had genital involvement, 35 patients (41.2%) presented with nail psoriasis, and 21 patients (24.7%) had palmoplantar disease. Among those with at least moderate psoriasis in these regions, 40 patients (47.1%) had sc-PGA ≥ 3, 16 patients (18.8%) had sPGA-G ≥ 3, 20 patients (23.5%) had f-PGA ≥ 3, and 9 patients (10.6%) had pp-PGA ≥ 3.

**Figure 1:**
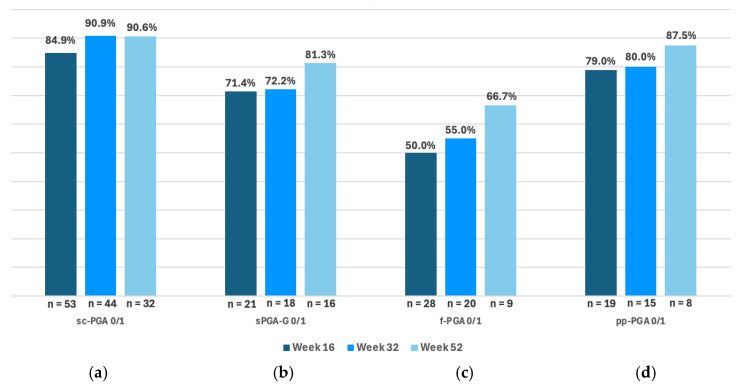
Percentage of patients achieving sc-PGA, sPGA-G, pp-PGA, and f-PGA of 0 or 1 (clear and almost clear) at weeks 16, 32 and 52. “n” represents the number of patients who reached weeks 16, 32, and 52, respectively. Abbreviation: sc-PGA: scalp-specific Physician’s Global Assessment; sPGA-G: static Physician’s Global Assessment of Genitalia; f-PGA: fingernail PGA; pp-PGA: palmoplantar PGA.

Concerning the cohort patients with scalp psoriasis, data at weeks 16, 32, and 52 were available for 53, 44, and 32 patients, respectively. A sc-PGA score of 0/1 was achieved in 84.9% (N = 45) (95% CI 72.4–93.3), 90.9% (N = 40) (95% CI 78.3–97.5), and 90.6% (N = 29) (95% CI 75.0–98.0) at weeks 16, 32, and 52, respectively, (Figure 1a). Among patients with genital psoriasis, sPGA-G score of 0/1 was achieved in 71.4% (N = 15) (95% CI 47.8–88.7) at week 16, 72.2% (N = 13) (95% CI 46.5–90.3) at week 32, and 81.3% (N = 13) (95% CI 54.4–96.0) at week 52 (Figure 1b). Regarding patients with nail psoriasis, f-PGA score of 0/1 was achieved in 50.0% (N = 14) (95% CI 30.6–69.4), 55.0% (N = 11) (95% CI 31.5–76.9), and 66.7% (N = 6) (95% CI 29.9–92.5) at weeks 16, 32, and 52, respectively, (Figure 1c). Finally, among patients with palmoplantar psoriasis, pp-PGA score of 0/1 was achieved in 79.0% (N = 15) (95% CI 54.4–93.9) at week 16, 80.0% (N = 12) (95% CI 51.9–95.7) at week 32, and 87.5% (N = 7) (95% CI 47.3–99.7) at week 52 (Figure 1d).

**Figure 2:**
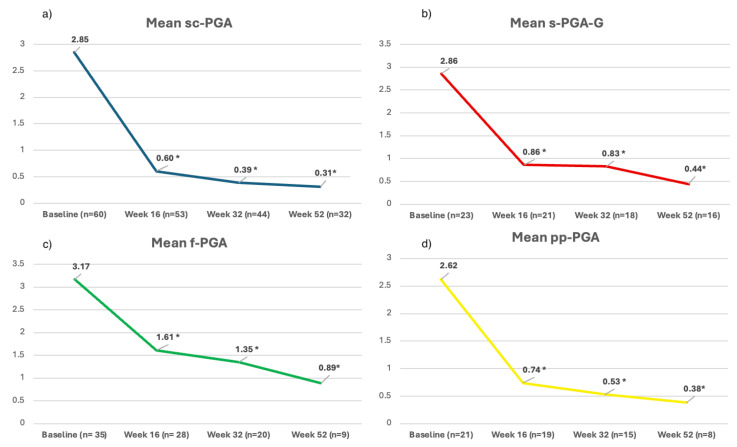
Mean site-specific sc-PGA (**a**); sPGA-G (**b**); f-PGA (**c**) and pp-PGA (**d**) of our population at baseline, week 16, week 32, and week 52. Abbreviations: sc-PGA: scalp-specific Physician’s Global Assessment; sPGA-G: static Physician’s Global Assessment of Genitalia; f-PGA: fingernail PGA; pp-PGA: palmoplantar PGA; * *p*-value < 0.001.

Moreover, mean site-specific PGA scores progressively decreased from baseline across all follow-up visits. The mean sc-PGA decreased from 2.85 at baseline to 0.60 at week 16, 0.39 at week 32, and 0.31 at week 52 (Figure 2a).

Regarding genital psoriasis, follow-up data for this cohort were available for 21, 18, and 16 patients at each follow-up visit (week 16, week 32, and week 52). In this subpopulation, the mean sPGA-G decreased from 2.86 at baseline to 0.86, 0.83, and 0.44 at weeks 16, 32, and 52, respectively (Figure 2b). Nail involvement was present in 45, 28, 20, and 9 patients at weeks 0, 16, 32, and 52, respectively. Nail psoriasis showed a progressive improvement, with the mean f-PGA decreasing from 3.17 at baseline to 1.61, 1.35, and 0.89 at weeks 16, 32, and 52, respectively (Figure 2c). Finally, palmo-plantar psoriasis was reported by 21 patients at baseline, with 19, 15, and 8 of them completing 16, 32, and 52 weeks of continuous treatment, respectively. A significant reduction was also observed in palmoplantar disease, with the mean pp-PGA decreasing from 2.62 at baseline to 0.74 at week 16, 0.53 at week 32, and 0.38 at week 52 (Figure 2d).

**Figure 3:**
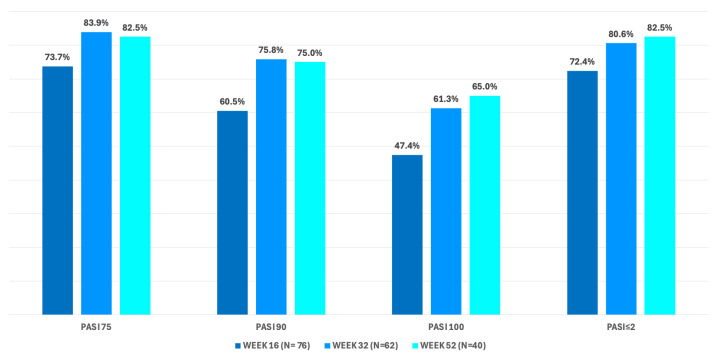
Percentages of patients achieving PASI75, PASI90 and PASI100 at weeks 16, 32 and 52. Abbreviation: PASI: Psoriasis Area and Severity Index.

In terms of overall effectiveness based on PASI reduction (Figure 3), PASI75, PASI90, and PASI100 responses were achieved at week 16 by 73.7% (95% CI 62.3–83.1), 60.5% (95% CI 48.6–71.6), and 47.4% (95% CI 35.8–59.2) of patients, respectively. We observed continuous improvement during the study, as at week 32 the same endpoints were achieved by 83.9% (95% CI 72.3–92.0), 75.8% (95% CI 63.3–85.8), and 61.3% (95% CI 48.1–73.4) of patients, respectively. Finally, at week 52 PASI75, PASI90, PASI100 were reached by 82.5% (95% CI 67.2–92.7), 75.0% (95% CI 58.8–87.3), and 65.0% (95% CI 48.3–79.4) of those who had completed one year of treatment. Considering the achievement of an absolute PASI ≤ 2, this outcome was reached by 72.4%, 80.6%, and 82.5% of patients at weeks 16, 32, and 52.

Regarding safety, adverse events (AEs) were reported in the cohort as follows (Table 2): 10 cases (11.8%) of oral candidiasis, 3 cases (3.5%) of eczematous dermatitis, 1 case (1.2%) of esophageal candidiasis, and 1 case (1.2%) of persistent gastrointestinal symptoms. Most adverse events were mild; however, 6 patients (7.1%) discontinued treatment due to AEs: 2 due to recurrent, treatment-resistant oral candidiasis; 2 due to extensive steroid-resistant eczematous dermatitis; 1 due to resistant esophageal candidiasis; and 1 due to persistent and worsening gastrointestinal symptoms. In terms of severity, the five AEs that led to treatment discontinuation can be classified as moderate, while all others were considered mild. Regarding management, mild oral candidiasis was treated with simple local and systemic antifungal therapies, whereas eczematous reactions were managed with topical corticosteroids, topical calcineurin inhibitors, and systemic corticosteroid therapy.

## 4. Discussion

Increasing knowledge of psoriasis pathogenesis has highlighted its systemic involvement, with significant implications not only for the skin but also for joint and cardiometabolic profile [1,14]. In addition to the classic plaque-type form affecting the extensor surfaces, several sites defined as difficult-to-treat areas, such as the scalp, genital region, nails, and palms/soles, may also be involved. These locations are not only more resistant to conventional therapies but also more considerably affect patients’ quality of life, emphasizing the need for more effective treatment strategies [3,6,7].

The advent of biologic therapies has markedly improved the management of psoriasis, including these challenging sites. Among the biologic agents, monoclonal antibodies targeting IL-17 have shown remarkable efficacy [11,13]. In particular, bimekizumab, a dual inhibitor of IL-17A and IL-17F, has emerged as a promising option in terms of both skin clearance and safety [16]. Pivotal trials demonstrated its strong efficacy against placebo (BE READY) and in direct comparison with other biologics, such as adalimumab (BE SURE), ustekinumab (BE VIVID), and secukinumab (BE RADIANT). Of note, these studies established more ambitious primary endpoints, moving beyond the traditional PASI 75 to PASI 90 and even PASI 100, thereby underscoring the rapid and profound efficacy of bimekizumab in psoriasis [19,22,23,24]. Supporting these observations, in the BE VIVID trial, which compared bimekizumab with placebo and ustekinumab (anti–IL-12/23 monoclonal antibody), 85% of patients who initiated treatment with bimekizumab achieved PASI90 and 59% achieved PASI100 as early as week 16, compared with 53% and 21%, respectively, in the ustekinumab arm [19]. Furthermore, in the BE RADIANT trial, which compared bimekizumab with secukinumab (an IL-17A inhibitor), after 48 weeks of follow-up, 74.8% of patients receiving bimekizumab reached PASI100, compared with 52.8% of those treated with secukinumab [23]. Even more compelling was the observation that, in this study, patients initially treated with secukinumab who were switched to bimekizumab subsequently achieved PASI100 in 76.6% of cases after 48 additional weeks, confirming a significant efficacy gap between the two agents [23].

However, to fully define the therapeutic potential of this monoclonal antibody, real-world data are essential. Gargiulo et al. [25] reported on 237 patients treated with bimekizumab, observing PASI 90, PASI 100, and absolute PASI ≤ 2 responses in 89.5%, 75.4%, and 94.7% of patients, respectively, already at week 16 [25]. These findings are broadly consistent with the outcomes observed for the respective endpoints in our cohort at comparable follow-up time points, although the overall proportion of patients achieving these targets was lower. This discrepancy is likely due to the fact that our study population primarily included patients with more treatment-resistant forms of psoriasis, frequently involving anatomical sites previously defined as difficult-to-treat. Such localizations are known to negatively influence therapeutic responsiveness, and therefore the attainment of stringent endpoints such as PASI90 and PASI100—which reflect complete or near-complete disease clearance across all affected areas—becomes inherently more challenging when compared with populations lacking involvement of these specific sites.

Difficult-to-treat areas have also been specifically evaluated in clinical trial sub-analyses. Long-term data from pivotal studies and their open-label extensions (BE BRIGHT and BE RADIANT OLE) confirmed sustained efficacy: among 821 patients with at least moderate scalp psoriasis (sc-PGA ≥ 3) at baseline, complete clearance (sc-PGA = 0) was achieved by 83.7%, 86.4%, and 85.9% of patients at weeks 16, 52, and 104, respectively. For nail disease, among 337 patients with baseline modified Nail Psoriasis Severity Index (mNAPSI) > 10, complete resolution (mNAPSI = 0) was reached by 63.4% and 68.5% of patients at weeks 52 and 104. In palmoplantar psoriasis, 193 patients with baseline pp-PGA ≥ 3 achieved pp-PGA = 0 in 87.4%, 88.3%, and 89.8% of cases at weeks 16, 52, and 104 [20,26]. Our findings are consistent with these results. At week 52, 90.6% of our patients achieved sc-PGA 0/1, 66.7% achieved f-PGA 0/1, and 87.5% achieved pp-PGA 0/1, confirming the strong effectiveness of bimekizumab in these difficult-to-treat locations.

Other real-world studies further support these observations. Campione et al. [27] reported that 57.0% of their 232 patients with nail psoriasis achieved f-PGA 0 as early as week 16, highlighting, in line with our results, the rapid activity of bimekizumab even in nails, which are typically slower to respond [27]. Data on genital psoriasis are also encouraging, according to various studies in the literature [28]. In particular, Hagino et al. [29] showed that among 36 patients with baseline sPGA-G ≥ 2, 95.7% achieved sPGA-G 0/1 at week 52 [28]. The slightly higher response rate compared to our study likely reflects the inclusion of patients with less severe baseline genital disease in their cohort. In the same study, scalp psoriasis also showed excellent responses, with 97.2% of patients achieving sc-PGA 0/1 at week 52 [29].

To date, systematic real-world data on palmoplantar psoriasis remain scarce, particularly with long-term follow-up, making our findings at 52 weeks highly relevant to establishing the effectiveness of bimekizumab in this area. However. The very limited sample size for this subgroup is a relevant limitation, and these findings should be interpreted cautiously.

In addition to the absolute rates of complete or near-complete clearance observed across the different areas, the mean PGA values reported in our study are also highly relevant. Achieving mean scores at week 52 of 0.31 for sc-PGA, 0.89 for f-PGA, 0.44 for sPGA-G, and 0.38 for pp-PGA reflects a substantial clinical improvement in sites that profoundly affect patients’ daily lives and overall quality of life. Our findings support the effectiveness of bimekizumab in treating complex patients with involvement of difficult-to-treat areas, consistent with current real-world evidence on other IL-17 drugs for plaque psoriasis [30].

Regarding safety, the spectrum and frequency of adverse events in our cohort were consistent with those reported in recent real-world experiences. For example, Megna et al. [31] observed oral candidiasis in 7.0% of 371 patients treated with bimekizumab for 16 weeks, comparable to the 11.8% observed in our study, taking into account our smaller sample size [31]. Overall, most adverse events were mild, and discontinuations were limited.

This study has a few limitations that should be acknowledged. First, one of the main limitations of our study is its retrospective single-center design, which does not allow the retrieval of missing data and limits the generalization of our findings, also because of the demographic characteristics of our population. Second, patient-reported outcomes such as the Dermatology Life Quality Index (DLQI) or itch scores were not consistently collected, preventing a comprehensive evaluation of quality-of-life improvements. Third, due to the real-world setting, some clinical parameters were missing at specific time points. Fourth, another limitation is the relatively small number of patients available for analysis at week 52 in certain subgroups, such as those with palmoplantar involvement. Thus, our findings for some categories (palmo-plantar psoriasis, in particular) should be interpreted with caution, given the smaller numbers. Fifth, the evaluation of the reported efficacy data is limited by the relatively small sample size, which does not allow for adequate patient stratification and multivariate analyses. Finally, a further limitation concerns the choice of site-specific PGAs to assess psoriasis severity, rather than scores such as the mNAPSI, which has been used in clinical trials to evaluate nail involvement but is less feasible in retrospective, real-world analyses. In support of this, several retrospective studies in the literature have employed the f-PGA to assess nail involvement, confirming its validity and reproducibility in a real-world setting [7]. Nevertheless, given the absence of real-world data with this length of follow-up, our findings remain clinically relevant for the use of bimekizumab in this area.

## 5. Conclusions

The results of our study confirm the effectiveness of bimekizumab across all difficult-to-treat areas analyzed. Notably, in palmoplantar psoriasis, this is a real-world evidence at 52 weeks demonstrating satisfying effectiveness. These findings support the consideration of bimekizumab as one of the possible primary therapeutic options for patients with psoriasis involving these challenging sites. Furthermore, the reported safety data raise no particular concerns regarding the long-term maintenance of clinical responses. Further investigation into the unique dual inhibition of IL-17A and IL-17F and its relationship with the high effectiveness of bimekizumab in these difficult-to-treat areas may help optimize its therapeutic potential. Lastly, we believe that future multicenter prospective registries will be essential to confirm these findings and further characterize the long-term safety of dual IL-17A/F blockade.

## Figures and Tables

**Table 1:** Demographic characteristics and disease severity scores at baseline of our population.

	Mean (SD)
Age, years	48.1 (16.8)
BMI, kg/m^2^	26.9 (5.8)
Disease duration, years	17.2 (13.7)
PASI baseline	13.2 (10.2)
	**N (%)**
Number of patients	85 (100.0)
Males	61 (71.8)
PsA	8 (9.4)
Scalp involvement	60 (70.6)
Genitalia involvement	23 (27.1)
Fingernails involvement	35 (41.2)
Palmoplantar involvement	21 (24.7)
sc-PGA ≥ 3	40 (47.1)
sPGA-G ≥ 3	16 (18.8)
f-PGA ≥ 3	20 (23.5)
pp-PGA ≥ 3	9 (10.6)
At least one cardiometabolic comorbidity	31 (36.5)
Bio-experienced	26 (30.6)

Abbreviation: BMI: Body Mass Index; PsA: Psoriatic Arthritis; PASI: Psoriasis Area and Severity Index; sc-PGA: scalp-specific Physician’s Global Assessment; sPGA-G: static Physician’s Global Assessment of Genitalia; f-PGA: fingernail PGA; pp-PGA: palmoplantar PGA.

**Table 2:** Reported adverse events during the treatment with bimekizumab in our cohort.

AEs	*N* (% on Total Population)
Oral candidiasis	10 (11.8%)
Eczematous dermatitis	3 (3.5%)
Esophageal candidiasis	1 (1.2%)
GI symptoms	1 (1.2%)

AE: adverse event.

## Data Availability

Data are available on request from the authors.

## Abbreviations

The following abbreviations are used in this manuscript:

BMI	Body Mass Index
PsA	Psoriatic Arthritis
PASI	Psoriasis Area and Severity Index
sc-PGA	scalp-specific Physician’s Global Assessment
sPGA-G	static Physician’s Global Assessment of Genitalia
f-PGA	fingernail PGA
pp-PGA	palmoplantar PGA
mNAPSI	modified Nail Psoriasis Severity Index
AE	adverse event.
CI	confidence interval

## References

[1] Armstrong A.W., Read C. (2020). Pathophysiology, Clinical Presentation, and Treatment of Psoriasis: A Review. JAMA.

[2] Lanna C., Galluzzi C., Zangrilli A., Bavetta M., Bianchi L., Campione E. (2022). Psoriasis in difficult to treat areas: Treatment role in improving health-related quality of life and perception of the disease stigma. J. Dermatol. Treat..

[3] Cannizzaro M.V., Coscarella G., Chiricozzi A. (2023). Brodalumab in the Treatment of Plaque Psoriasis Localized in Difficult-to-Treat Areas: A Narrative Review. Dermatol. Pract. Concept..

[4] Wozel G. (2008). Psoriasis treatment in difficult locations: Scalp, nails, and intertriginous areas. Clin. Dermatol..

[5] Armstrong A.W., Bagel J., Van Voorhees A.S., Robertson A.D., Yamauchi P.S. (2015). Combining biologic therapies with other systemic treatments in psoriasis: Evidence-based, best-practice recommendations from the Medical Board of the National Psoriasis Foundation. JAMA Dermatol..

[6] Kivelevitch D., Frieder J., Watson I., Paek S.Y., Menter M.A. (2018). Pharmacotherapeutic approaches for treating psoriasis in difficult-to-treat areas. Expert. Opin. Pharmacother..

[7] Orsini D., Gargiulo L., Ibba L., Cascio Ingurgio R., Valenti M., Perugini C., Pacifico A., Maramao F.S., Frascione P., Costanzo A. (2023). Effectiveness of risankizumab in plaque psoriasis with involvement of difficult-to-treat areas: A real-world experience from two referral centers. J. Dermatol. Treat..

[8] Gisondi P., Altomare G., Ayala F., Bardazzi F., Bianchi L., Chiricozzi A., Costanzo A., Conti A., Dapavo P., De Simone C. (2017). Italian guidelines on the systemic treatments of moderate-to-severe plaque psoriasis. J. Eur. Acad. Dermatol. Venereol..

[9] García-Patiño M.G., Marcial-Medina M.C., Ruiz-Medina B.E., Licona-Limón P. (2024). IL-17 in skin infections and homeostasis. Clin. Immunol..

[10] Ghoreschi K., Balato A., Enerbäck C., Sabat R. (2021). Therapeutics targeting the IL-23 and IL-17 pathway in psoriasis. Lancet.

[11] Brembilla N.C., Boehncke W.H. (2023). Revisiting the interleukin 17 family of cytokines in psoriasis: Pathogenesis and potential targets for innovative therapies. Front. Immunol..

[12] Armstrong A.W., Blauvelt A., Callis Duffin K., Huang Y.H., Savage L.J., Guo L., Merola J.F. (2025). Psoriasis. Nat. Rev. Dis. Primers..

[13] Yiu Z.Z., Griffiths C.E. (2016). Interleukin 17-A inhibition in the treatment of psoriasis. Expert. Rev. Clin. Immunol..

[14] Glatt S., Baeten D., Baker T., Griffiths M., Ionescu L., Lawson A.D.G., Maroof A., Oliver R., Popa S., Strimenopoulou F. (2018). Dual IL-17A and IL-17F neutralisation by bimekizumab in psoriatic arthritis: Evidence from preclinical experiments and a randomised placebo-controlled clinical trial that IL-17F contributes to human chronic tissue inflammation. Ann. Rheum. Dis..

[15] Kolbinger F., Loesche C., Valentin M.A., Jiang X., Cheng Y., Jarvis P., Peters T., Calonder C., Bruin G., Polus F. (2017). β-Defensin 2 is a responsive biomarker of IL-17A-driven skin pathology in patients with psoriasis. J. Allergy Clin. Immunol..

[16] Kokolakis G., Ghoreschi K. (2022). The Clinical Significance of Simultaneous IL-17A and IL-17F Blockade in Psoriasis Non-Responding to Anti-IL17A Therapy. J. Clin. Med..

[17] Egeberg A., Andersen Y.M.F., Halling-Overgaard A.S., Alignahi F., Thyssen J.P., Burge R., Mallbris L. (2020). Systematic review on rapidity of onset of action for interleukin-17 and interleukin-23 inhibitors for psoriasis. J. Eur. Acad. Dermatol. Venereol..

[18] Blauvelt A., Langley R.G., Lebwohl M., Strober B., Warren R.B., Puig L., Morita A., Gordon K.B., Fernandez-Peñas P., Kavanagh S. (2025). Bimekizumab durability of efficacy through 196 weeks and safety through 4 years in patients with moderate to severe plaque psoriasis: Results from the BE BRIGHT open-label extension trial. J. Am. Acad. Dermatol..

[19] Reich K., Papp K.A., Blauvelt A., Langley R.G., Armstrong A., Warren R.B., Gordon K.B., Merola J.F., Okubo Y., Madden C. (2021). Bimekizumab versus ustekinumab for the treatment of moderate to severe plaque psoriasis (BE VIVID): Efficacy and safety from a 52-week, multicentre, double-blind, active comparator and placebo controlled phase 3 trial. Lancet.

[20] Merola J.F., Gottlieb A.B., Pinter A., Elewski B., Gooderham M., Warren R.B., Piaserico S., Wixted K., Cross N., Tilt N. (2024). Bimekizumab Efficacy in High-Impact Areas: Pooled 2-Year Analysis in Scalp, Nail, and Palmoplantar Psoriasis from Phase 3/3b Randomized Controlled Trials. Dermatol. Ther..

[21] Gordon K.B., Langley R.G., Warren R.B., Okubo Y., Rosmarin D., Lebwohl M., Peterson L., Madden C., de Cuyper D., Davies O. (2024). Bimekizumab safety in patients with moderate-to-severe plaque psoriasis: Pooled data from up to 3 years of treatment in randomized phase III trials. Br. J. Dermatol..

[22] Gordon K.B., Foley P., Krueger J.G., Pinter A., Reich K., Vender R., Vanvoorden V., Madden C., White K., Cioffi C. (2021). Bimekizumab efficacy and safety in moderate to severe plaque psoriasis (BE READY): A multicentre, double-blind, placebo-controlled, randomised withdrawal phase 3 trial. Lancet.

[23] Strober B., Paul C., Blauvelt A., Thaçi D., Puig L., Lebwohl M., White K., Vanvoorden V., Deherder D., Gomez N.N. (2023). Bimekizumab efficacy and safety in patients with moderate to severe plaque psoriasis: Two-year interim results from the open-label extension of the randomized BE RADIANT phase 3b trial. J. Am. Acad. Dermatol..

[24] Thaçi D., Vender R., de Rie M.A., Conrad C., Pariser D.M., Strober B., Vanvoorden V., Wang M., Madden C., de Cuyper D. (2023). Safety and efficacy of bimekizumab through 2 years in patients with moderate-to-severe plaque psoriasis: Longer-term results from the BE SURE randomized controlled trial and the open-label extension from the BE BRIGHT trial. Br. J. Dermatol..

[25] Gargiulo L., Narcisi A., Ibba L., Balato A., Bianchi L., Brianti P., Buononato D., Burlando M., Caldarola G., Campanati A. (2023). Effectiveness and safety of bimekizumab for the treatment of plaque psoriasis: A real-life multicenter study-IL PSO (Italian landscape psoriasis). Front. Med..

[26] Strober B., Tada Y., Mrowietz U., Lebwohl M., Foley P., Langley R.G., Warrenm R.B., Wang M., Vanvoorden V., Szilagyi B. (2023). Bimekizumab maintenance of response through 3 years in patients with moderate-to-severe plaque psoriasis: Results from the BE BRIGHT open-label extension trial. Br. J. Dermatol..

[27] Campione E., Artosi F., Shumak R.G., Giunta A., Argenziano G., Assorgi C., Balato A., Bernardini N., Brunasso A.M.G., Burlando M. (2024). Fast Clinical Response of Bimekizumab in Nail Psoriasis: A Retrospective Multicenter 36-Week Real-Life Study. Pharmaceuticals.

[28] Orsini D., Malagoli P., Balato A., Bianchi L., Brianti P., Buononato D., Burlando M., Caldarola G., Campanati A., Campione E. (2024). Bimekizumab for the Treatment of Plaque Psoriasis with Involvement of Genitalia: A 16-Week Multicenter Real-World Experience—IL PSO (Italian Landscape Psoriasis). Dermatol. Pract. Concept..

[29] Hagino T., Saeki H., Fujimoto E., Kanda N. (2025). Bimekizumab for the treatment of genital, scalp, and nail psoriasis: A 52-week real-world study. J. Dermatol..

[30] Valenti M., Gargiulo L., Ibba L., Malagoli P., Amoruso F., Balato A., Bardazzi F., Burlando M., Carrera C.G., Dapavo P. (2024). Long-Term Effectiveness and Safety of Ixekizumab for the Treatment of Moderate-to-Severe Plaque Psoriasis: A Five-Year Multicenter Retrospective Study-IL PSO (Italian Landscape Psoriasis). Dermatol. Ther..

[31] Megna M., Orsini D., Assorgi C., Balato A., Balestri R., Bianchi L., Brianti P., Brunasso G., Buononato D., Burlando M. (2025). The risk of candidiasis during treatment with bimekizumab for the management of plaque psoriasis: A 16-week multicentre real-world experience—IL PSO (Italian Landscape Psoriasis). Clin. Exp. Dermatol..

